# The ATPase TER94 regulates Notch signaling during *Drosophila* wing development

**DOI:** 10.1242/bio.038984

**Published:** 2018-12-10

**Authors:** Yunlong Li, Tong Liu, Junzheng Zhang

**Affiliations:** Department of Entomology and MOA Key Lab of Pest Monitoring and Green Management, College of Plant Protection, China Agricultural University, 100094 Beijing, China

**Keywords:** Notch, TER94, *Drosophila*, Wing development

## Abstract

The evolutionarily conserved Notch signaling pathway plays crucial roles in various developmental contexts. Multiple mechanisms are involved in the regulation of Notch pathway activity. Identified through a genetic mosaic screen, we show that the ATPase TER94 acts as a positive regulator of Notch signaling during *Drosophila* wing development. Depletion of TER94 causes marginal notches in the adult wing and the reduction of Notch target genes *wingless* and *cut* during wing margin formation. We provide evidence that TER94 is likely required for proper Notch protein localization and activation. Furthermore, we show that knockdown of the TER94 adaptor p47 leads to similar Notch signaling defects. Although the TER94 complex is implicated in various cellular processes, its role in the regulation of Notch pathways was previously uncharacterized. Our study demonstrates that TER94 positively regulates Notch signaling and thus reveals a novel role of TER94 in development.

This article has an associated First Person interview with the first author of the paper.

## Abstract (precis)

**Summary:** Our study demonstrates that the ATPase TER94 and the p47 adaptor positively regulate Notch signaling during *Drosophila* wing development, thus establishing a functional interaction between TER94 and Notch signaling activity.

## INTRODUCTION

The Notch signaling pathway plays important roles in tissue development and homeostasis by regulating multiple biological processes such as cell fate determination, cell proliferation and cell cycle progression (Bray, 2006). Given the importance of Notch signaling in various cell types, it is unsurprising that aberrant Notch signaling in humans leads to many malignant diseases, such as cancers and neurological disorders (Alberi et al., 2013; Aster et al., 2017). The canonical Notch signaling cascade operates in a cell–cell contact dependent manner with a small number of core components (Andersson et al., 2011). Upon binding to its ligands Delta (Dl) and/or Serrate, the Notch receptor undergoes a series of proteolytic cleavages which results in the generation of the Notch intracellular domain (NICD) (Guruharsha et al., 2012). The NICD translocates into the nucleus, where it associates with the transcription factor Suppressor of Hairless [Su(H)] and the co-activator Mastermind to stimulate the expression of downstream target genes (Bray, 2006). In the absence of active NICD, Su(H) recruits transcription suppressors to inhibit Notch targets expression (Andersson et al., 2011; Guruharsha et al., 2012). Numerous auxiliary proteins are found to fine-tune the Notch pathway at different signal transduction steps, including intracellular trafficking of Notch receptor and ligands, production of the receptor and ligands as well as the proteolytic processing to generate active NICD (Andersson et al., 2011; Guruharsha et al., 2012). However, our understanding about how Notch signaling is regulated in a context dependent manner during development is still incomplete.

TER94 is an evolutionally conserved ATPase known for its sheer abundance and essential functions in eukaryotic cells (Meyer et al., 2012). It has been shown that TER94 plays crucial roles in ubiquitin related cellular activities such as protein degradation, cell cycle regulation, DNA replication and gene expression (Baek et al., 2013). More than forty TER94-interacting cofactors have been identified so far, and they are assembled in various combinations to achieve the functional diversity (Richly et al., 2005; Stach and Freemont, 2017). Recent studies suggest that TER94 is involved in the regulation of Hedgehog (Hh), Wingless (Wg) and Bone morphogenetic protein (BMP) signaling pathways in *Drosophila*. It has been demonstrated that TER94 regulates the partial degradation of Hh pathway transcription factor Cubitus interruptus (Zhang et al., 2013). TER94 antagonizes Wg signaling by promoting the transcription factor Armadillo to proteolytic degradation (Reim et al., 2014). In addition, TER94 is also required for maintaining BMP signaling during early embryogenesis (Zeng et al., 2014). These studies implicate that TER94 possesses regulatory potentials in developmental signaling pathways, most likely through affecting ubiquitin conjugation related processes on key signal transduction components. However, it is still unknown whether TER94 is involved in Notch signaling regulation during development.

During a genetic mosaic screen, we found that TER94 is required for wing margin formation in *Drosophila*. Furthermore, we find that loss of TER94 function downregulates Notch target genes’ expression. We provide evidence that TER94 and the p47 adaptor positively regulate Notch signaling during fly wing development.

## RESULTS

### TER94 regulates Notch signaling in the *Drosophila* wing

**Fig. 1.**
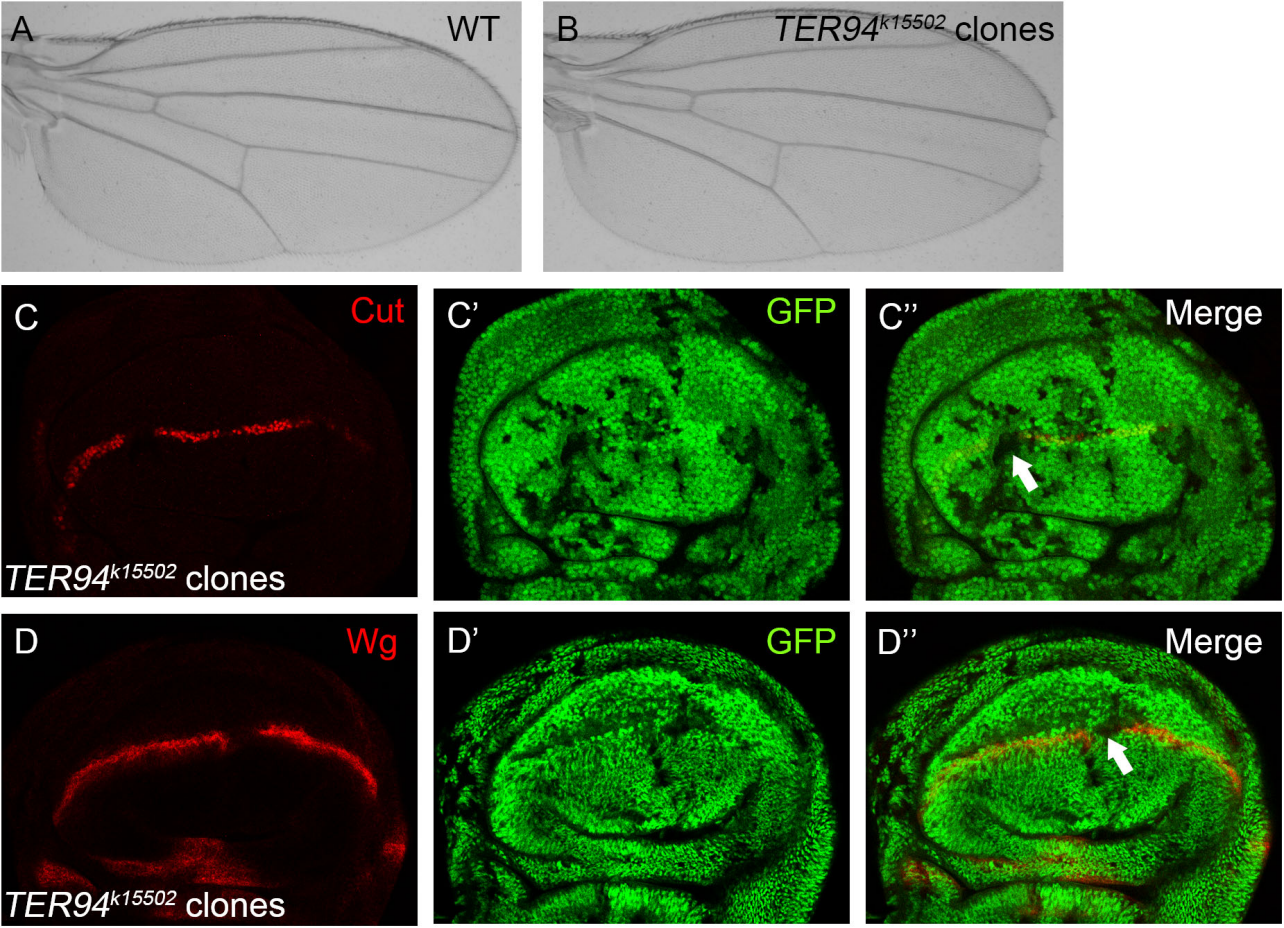
**TER94 is required for Notch** **signaling output in the fly wing.** (A–B) The adult wing of parental *Ubx-FLP*; *FRT42D*, *Ubi-GFP* stock is used as wild-type control (A). Wing margin loss is observed in fly wings harboring *TER94^k15502^* homozygous mutant clones (B). (C–D″) Expression of Notch signaling targets Cut (C) and Wg (D) are abolished in *TER94^k15502^* homozygous mutant clones (marked by absence of GFP). C′ and D′ show the green channel, C″ and D″ are the merged view of both channels. Representative mutant clones are indicated by arrows.

**Fig. 2.**
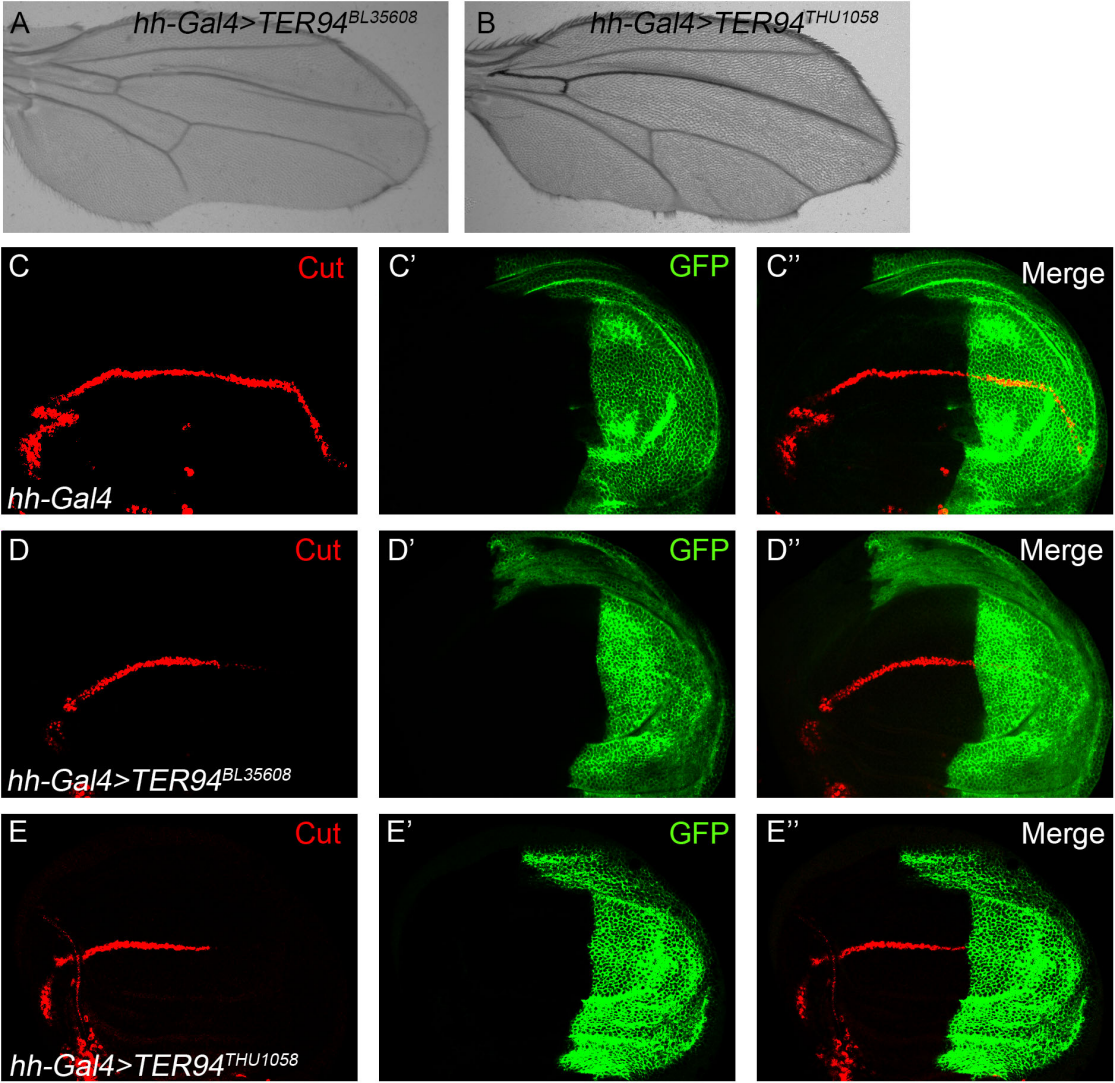
**RNAi knockdown of TER94** **dampens Notch signaling activity.** (A–B) Adult wings from flies expressing *UAS-TER94^BL35608^* (A) and *UAS-TER94^THU1058^* (B), two independent transgenic RNAi constructs under the control of *hh-Gal4* exhibit obvious marginal defects. (C–E″) In response to Notch signaling activation, Cut is expressed in cells along the D/V boundary of the developing wing disc (C). RNAi knockdown of TER94 driven by the *hh-Gal4* reduces Cut protein levels in the posterior compartment of the wing disc (D–E). GFP marks the expression domain of *hh-Gal4*. C′, D′ and E′ show the green channel. C″, D″ and E″ are the merged view of both channels.

We have performed a genetic mosaic screen to identify modifiers of Notch signaling in the *Drosophila* wing (Ren et al., 2018). During the screen we found that fly wings harboring homozygous *TER94^k15502^* mutant clones displayed wing margin nicking phenotypes (Fig. 1A–B). As the ‘nicked wing margin’ represents the classical phenotype associated with dysregulation of Notch signaling, we further investigated whether Notch signaling activity is affected in *TER94^k15502^* mutant cells. In the wild-type wing disc, Cut and Wg are produced in a narrow strip of cells at the dorso/ventral (D/V) boundary in response to Notch signaling (Fig. S1A–B). The expression of Cut and Wg were abolished when the *TER94^k15502^* mutant clones are located at the D/V boundary (Fig. 1C–D). To further confirm the requirement of TER94 in Notch signaling, we examined the effect of knockdown TER94 function by RNAi. Wing margin notches were observed when two independent RNAi lines were driven by the *hh-Gal4* to inhibit TER94 expression in the posterior compartment of the developing wing (Fig. 2A–B). In agreement with the adult wing phenotype, expression of the Notch target gene Cut was obviously reduced in TER94 RNAi cells (Fig. 2C–E). Collectively, these findings provide evidence that TER94 is required for activation of Notch downstream target genes during wing margin formation.

### The ATPase activity of TER94 is essential for Notch signaling regulation

**Fig. 3.**
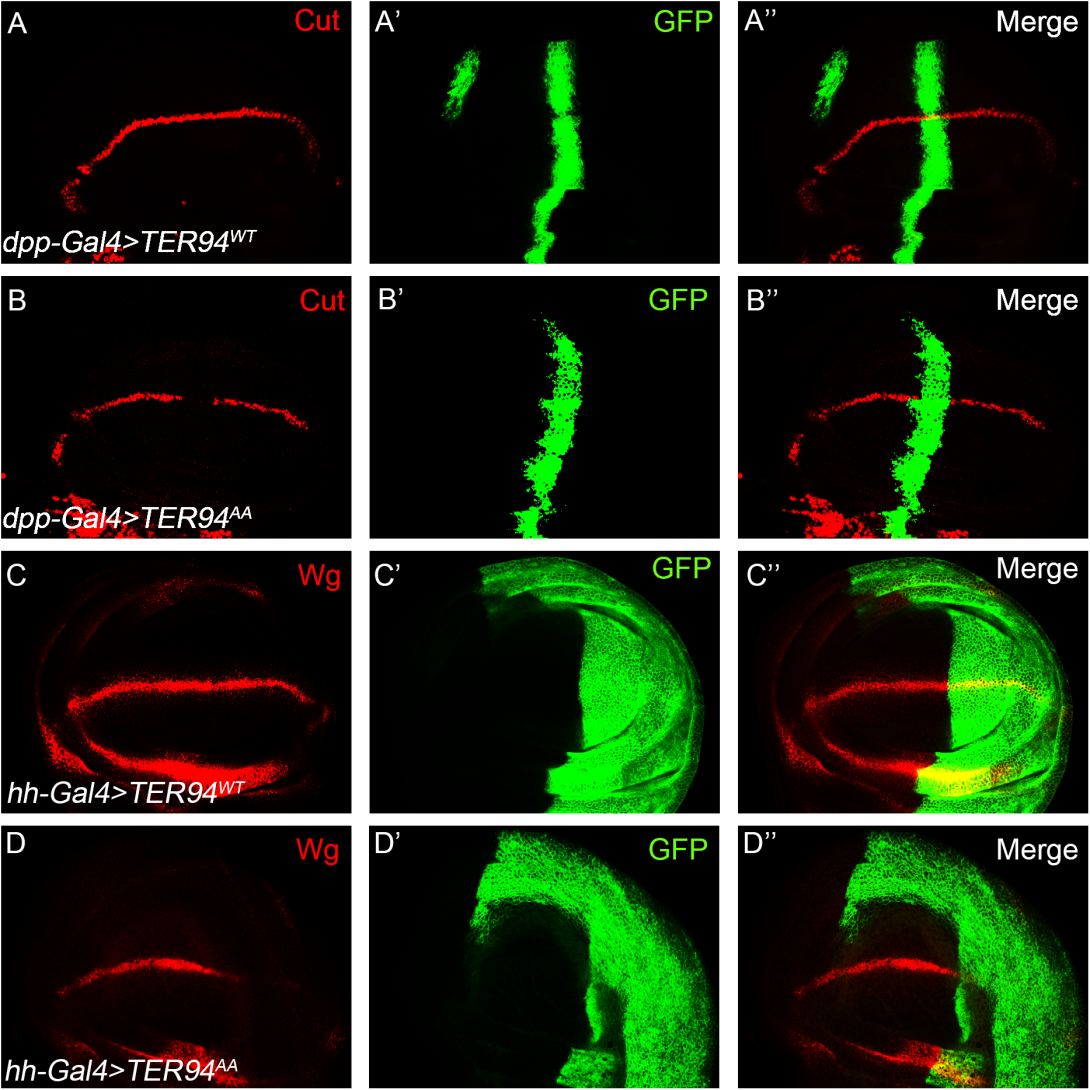
**The ATPase activity of TER94 is required** **for Notch signaling.** (A–B) The level of Cut protein is unaltered in TER94^WT^ expressing cells (A). In contrast, over-expression of TER94^AA^ significantly reduces Cut expression level in the wing disc (B). GFP marks the expression domain of *dpp-Gal4*. (C–D″) The expression pattern of Wg is not changed by over-expression of TER94^WT^ (C). Notably, TER94^AA^ eliminates Wg expression in the posterior compartment of the wing disc under the same condition. GFP marks the expression domain of *hh-Gal4*. A′, B′, C′ and D′ show the green channel. A″, B″, C″ and D″ are the merged view of both channels.

TER94 belongs to the family of ATPases associated with diverse cellular activities (AAA-ATPases), which often utilize their ATPase activity to convert the energy of ATP hydrolysis to structurally remodel client proteins (Meyer et al., 2012). To further understand the underlying biochemical basis, we examined whether the ATPase activity is essential for TER94 to modulate Notch signaling activity. Expression of the wild-type TER94 (TER94^WT^) was able to rescue the wing margin defects caused by RNAi knockdown (Fig. S2A–B). However, co-expression of the ATP-binding defective form of TER94 (TER94^AA^) (Zhang et al., 2013) with the RNAi resulted in larval lethality. In fact, over-expression of TER94^AA^, but not TER94^WT^, in the wild-type background sufficed to cause Notch signaling defects. Overexpression of TER94^AA^ in the developing wing under the control of *hh-Gal4* or *dpp-Gal4* resulted in a significant reduction of the Notch signaling target genes Cut (Fig. 3A–B) and Wg (Fig. 3C–D). These observations suggest that TER94^AA^ acts as a dominant-negative mutant and indicate that the ATPase activity of TER94 is essential for Notch signaling regulation.

### Ter94 likely regulates Notch protein in the signal receiving cells

**Fig. 4.**
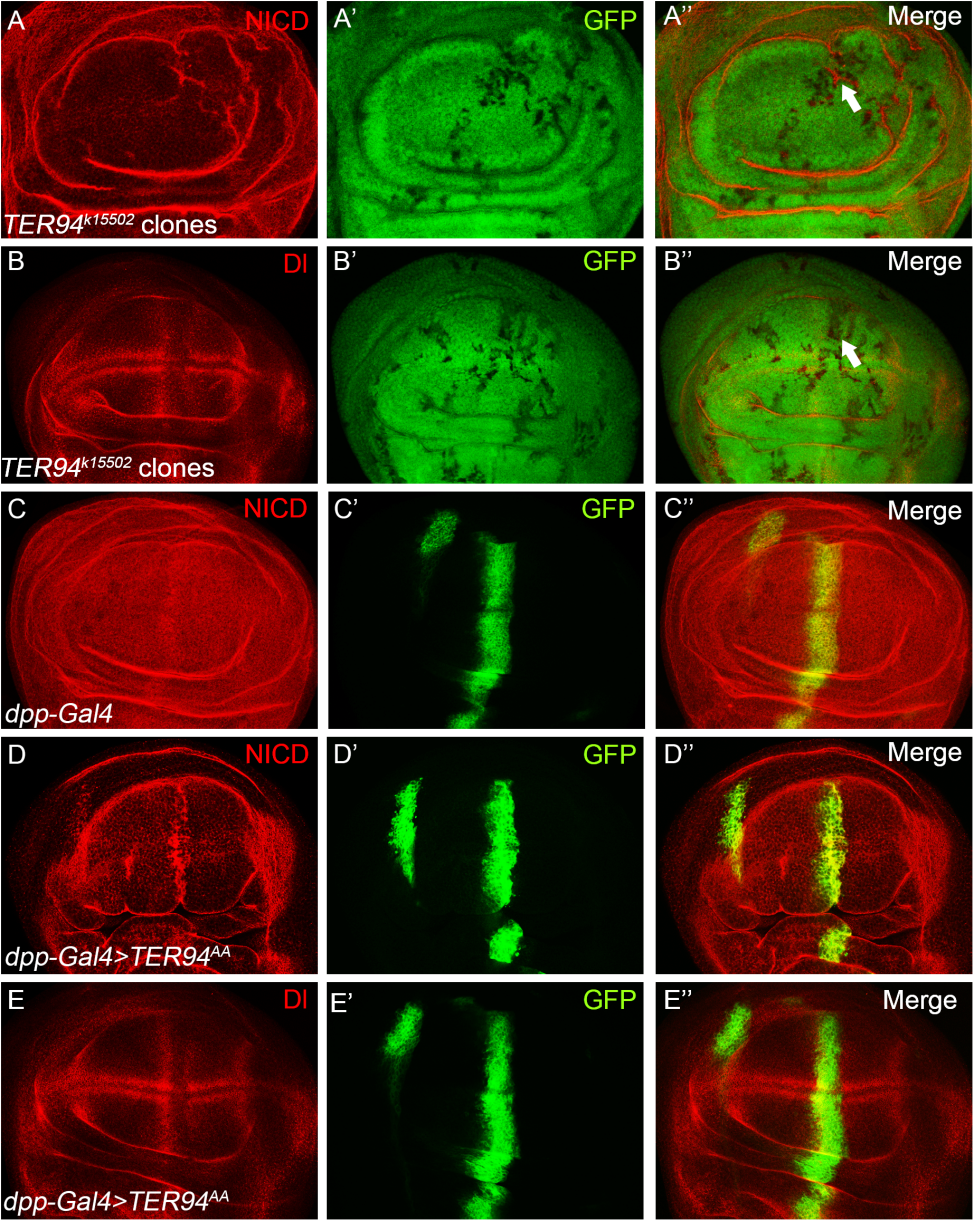
**TER94 modulates the Notch** **receptor protein.** (A–B″) The Notch receptor proteins are accumulated in *TER94^k15502^* mutant clones (A), while Dl proteins are largely unaffected in *TER94^k15502^* mutant clones (B). Clones are marked by the absence of GFP. Representative mutant clones are indicated by arrows. (C–E″) Disruption of TER94 function by over-expressing TER94^AA^ leads to accumulation of Notch (C–D), but not Dl (E) proteins in wing disc cells. GFP marks the expression domain of *dpp-Gal4*. A′, B′, C′, D′ and E′ show the green channel. A″, B″, C″, D″ and E″ are the merged view of both channels.

Having identified that TER94 is involved in regulating Notch signaling, we sought to further elucidate the signal transduction step targeted by TER94. We noticed that NICD was accumulated in the *TER94^k15502^* mutant clones (Fig. 4A), while Dl protein was largely unaffected (Fig. 4B). Similarly, over-expression of TER94^AA^ led to an aberrant accumulation of NICD, but did not affect Dl accumulation in wing disc cells (Fig. 4C–E). These results indicate that TER94 might function directly on the Notch receptor protein.

**Fig. 5.**
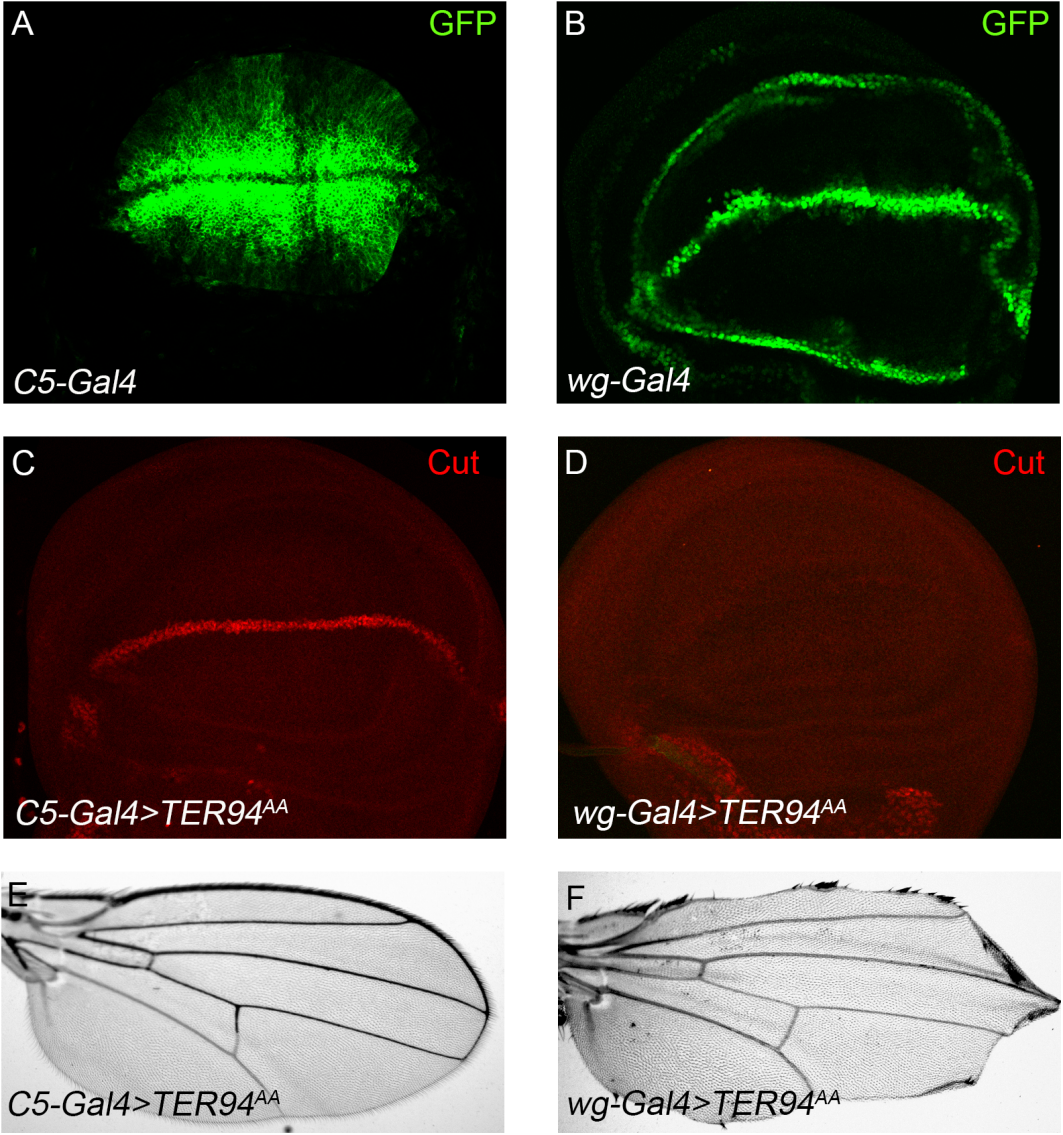
**TER94 promotes Notch signaling in the signal-receiving cells.** (A–B) Expression patterns of *C5-Gal4* and *wg-Gal4* driving GFP in wing disc. The *C5-Gal4* is active in D/V boundary flanking cells of the wing pouch (A), while *wg-Gal4* drives GFP expression along the D/V boundary (B). (C–F) Over-expression of TER94^AA^ by *C5-Gal4* does not affect the expression of Cut in the wing disc (C) and margin formation in the adult wing (E). When expressed in the signal receiving cells by the *wg-Gal4*, TER94^AA^ leads to diminishment of Cut expression (D) and loss of wing margin (F).

During fly wing margin formation, refinement of Notch and ligand expression through lateral inhibition results in the release of high levels of NICD along the D/V boundary, with the flanking cells containing high levels of ligands (Fig. S1C–D). Thus the Notch ligands activate down-stream targets in the signal receiving cells along the D/V boundary. We further used C5-Gal4 and wg-Gal4, to express TER94^AA^ in the complementary signal-sending and signal-receiving cells of wing imaginal disc. The C5-Gal4 is expressed in signal-sending cells flanking the D/V boundary (Fig. 5A), while wg-Gal4 is expressed in the active Notch signal receiving cells along the D/V boundary (Fig. 5B). By examining Notch target gene expression, we could determine in which cells TER94 is working to affect the Notch pathway (Hall et al., 2017). C5>TER94^AA^ appeared to have no effect on Cut expression (Fig. 5C). On the other hand, wg>TER94^AA^ gave very contrasting results. Disruption of TER94 function in the Notch receptor-expressing cells resulted in strong reduction of Cut (Fig. 5D). Consistently, wing margin was found to be intact in C5>TER94^AA^ adults (Fig. 5E) but nearly completely lost in wg>TER94^AA^ flies (Fig. 5F). We also noticed that TER94 RNAi driven by wg-Gal4 but not C5-Gal4 led to wing margin nicking phenotypes in the progeny (Fig. S2C–F). These findings support the notion that TER94 regulates Notch signaling in the signal responding cells, most likely through modifying Notch protein activity during fly wing development.

### TER94 complex might regulate Notch protein through the ubiquitin system

**Fig. 6.**
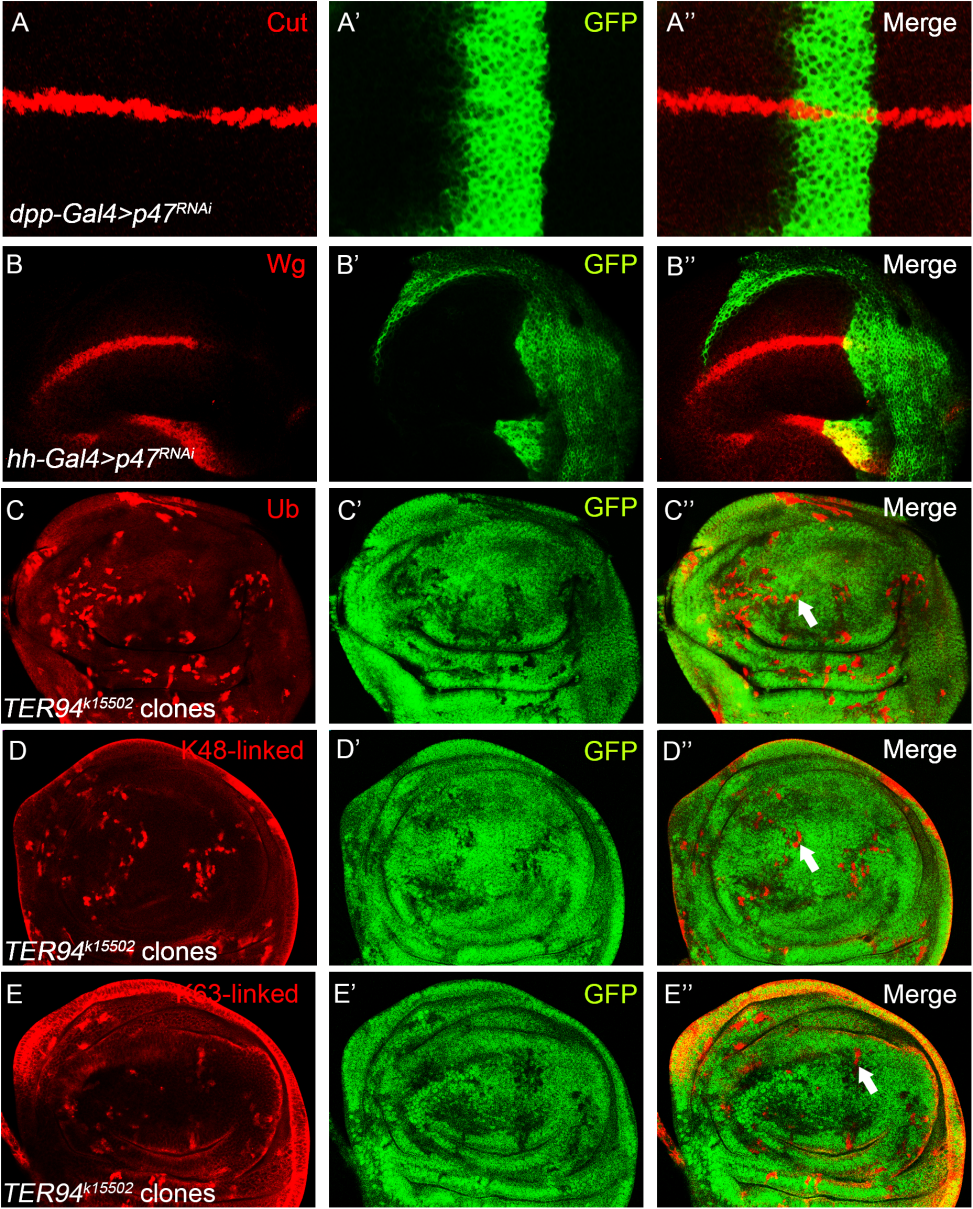
**The TER94-p47 complex is** **involved in Notch signaling regulation.** (A–B″) Knockdown of a TER94 co-factor p47 by RNAi results in reduction of Notch signaling targets Cut (A) and Wg (B) in the wing disc. GFP marks the expression domain of *dpp-Gal4* (A) and *hh-Gal4* (B). (C–E″) The TER94 complex regulates global ubiquitination status in wing disc cells. Levels of overall ubiquitin (C), K48-linkage ubiquitin chains (D) and K63-linkage ubiquitin chains (E) are accumulated in the *TER94^k15502^* mutant clones (marked by absence of GFP). A′, B′, C′, D′ and E′ show the green channel. A″, B″, C″, D″ and E″ are the merged view of both channels. Representative mutant clones are indicated by arrows.

TER94 forms core complexes with mutually exclusive major cofactors, and the diverse functions of TER94 are dictated by the adaptor proteins (Schuberth and Buchberger, 2008; van den Boom and Meyer, 2018). For example, TER94 forms a complex with its adaptors Ufd1-like and dNpl4 to regulate Ci degradation (Zhang et al., 2013), while it interacts with Alx/UbxN6 to regulate Wg signaling (Reim et al., 2014). We hypothesized that a specific TER94 cofactor might be required for the modulation of Notch signaling. Therefore, we performed a small scale RNAi screen to identify the TER94 adaptor involved in Notch signaling regulation (Table S1). Among the 12 potential TER94 adaptors we screened, we found that p47 is likely involved in Notch signaling regulation. Knockdown of p47 by RNAi caused the reduction of Notch targets Cut (Fig. 6A) and Wg (Fig. 6B) at the D/V boundary in the wing disc. These observations led us to speculate that p47 is a TER94 co-factor for regulation of Notch signaling.

**Fig. 7.**
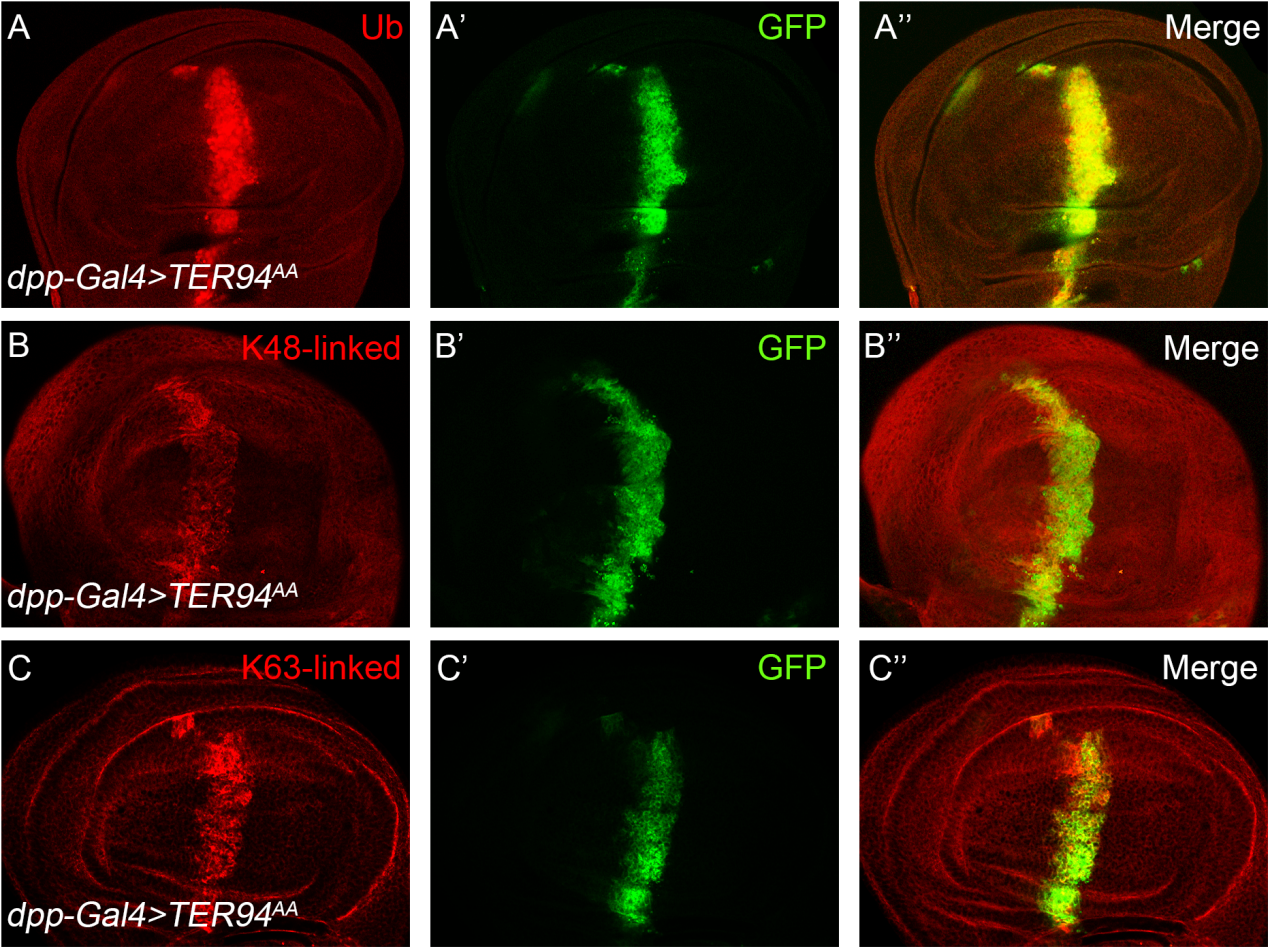
**The ATPase activity of** **TER94 is required for ubiquitin homeostasis.** Over-expression of TER94^AA^ affects the levels of overall ubiquitin (A), K48-linkage ubiquitin chains (B) and K63-linkage ubiquitin chains (C) in the wing disc cells. GFP marks the expression domain of *dpp-Gal4*. A′, B′ and C′ show the green channel. A″, B″ and C″ are the merged view of both channels.

Numerous studies have revealed that TER94 complexes bind to ubiquitinated substrate proteins and then promote remodeling of the substrates to facilitate downstream steps (Ye, 2006; Jentsch and Rumpf, 2007; Stolz et al., 2011). Indeed, the p47 cofactor protein contains an ubiquitin-associated (UBA) and an ubiquitin-X (UBX), both could recognize ubiquitin modified cargos (Uchiyama and Kondo, 2005). In agreement with the current model, we found that ubiquitinated proteins are highly accumulated in *TER94^k15502^* mutant cells (Fig. 6C). The fates of ubiquitinated protein substrates are often dictated by the ubiquitin chain linkages attached on them (Akutsu et al., 2016). It has been suggested that TER94 mainly regulates K11-linked ubiquitin chains in the wing disc cells (Zhang et al., 2013). Interestingly, we found that two other major types of ubiquitin linkages, the K48 and K63 linked ubiquitin chains were also accumulated in *TER94^k15502^* mutant cells (Fig. 6D,E). As a control, we showed here that all three forms of ubiquitin were ubiquitously distributed in the wild-type wing disc cells (Fig. S3). Similar results were obtained when TER94 function was disrupted by over-expressing the dominant negative TER94^AA^. The level of global ubiquitination (Fig. 7A), as well as K48 linked ubiquitin chains (Fig. 7B) and K63 linked ubiquitin chains (Fig. 7C) were significantly increased in TER94^AA^ overexpressing cells. These results suggest that TER94 is required for the homeostasis of cellular ubiquitin system, and might regulate Notch protein through the ubiquitin system during *Drosophila* wing development.

## DISCUSSION

The data presented here described the TER94 ATPase as a novel regulator of the Notch signaling pathway in *Drosophila*. Although *Drosophila* TER94 acts broadly to regulate the activity of Hh, Wg and BMP signaling pathways (Zhang et al., 2013; Reim et al., 2014), our report is the first to establish a functional interaction between TER94 and Notch activity during wing development. More specifically, we found that TER94 might regulate Notch protein activity in the signal receiving cells during wing margin formation. We showed that disruption of TER94 activity resulted in profound changes in Notch protein distribution, but had little effect on the ligand Dl. Using cell-type-specific Gal4 drivers, we further demonstrated that TER94 is required in the signal receiving cells for proper activation of Notch signaling. Intriguingly, we observed elevation of Notch protein level and inhibition of Notch signaling activity at the same time when TER94 function was disrupted. These results suggest that the accumulated Notch proteins in TER94 loss-of-function cells were incompetent for signaling activity. Notch proteins with abnormal intracellular localization or modification always accumulate to very high levels in different cellular compartments, but lead to severe inhibition of signaling activity (Okajima et al., 2005; Tien et al., 2008; Acar et al., 2008; Vaccari et al., 2010; Groth et al., 2013). As similar defects were observed for mutations affecting Notch protein folding, exiting from the ER and endocytosis from the plasma membrane, exactly which aspect is regulated by TER94 needs further investigation.

Given the central role of TER94 in a wide range of cellular activities, it is reasonable to assume that TER94 is involved in various developmental events. However, it was difficult to analyze the TER94 loss-of-function phenotypes because of it is essential for cell growth. Massively inhibiting TER94 function by RNAi and TER94^AA^ resulted in early lethality, while the size of *TER94^k15502^* mutant clones was found to be too small to display prominent phenotypes in adult tissues (Zhang et al., 2013; Reim et al., 2014). Interestingly, nicking of wing margin was readily observed in adults bearing *TER94^k15502^* clones (Fig. 1B). We have noticed that small sized mutant clones were able to cause the characteristic wing notch phenotype (Ren et al., 2018). The wing margin is formed by a narrow row of cells, 2–5 cells wide, along the D/V boundary. Therefore, mutant clones with a diameter of 2–5 cells are sufficient to induce marginal defects when they are located across the D/V boundary. As one of the earliest recorded mutant phenotypes in fly, nicked wing margin might still be the most reliable readout for Notch signaling defects in terms of sensitivity. Indeed, when we mildly inhibited TER94 function to avoid cell growth defects in the developing wing, marginal notches are consistently discovered (Figs 2A,B, 5E,F). Our data, however, does not exclude the roles of TER94 in other Notch regulated developmental processes nor other signaling pathways. The adult phenotypes are determined by the inhibition efficiency, as well as the cell types and developmental stages that TER94 RNAi and TER94^AA^ are applied in. Future studies are needed to examine TER94's function in a more spatial- and temporal-specific fashion, and the outcomes will help us to better understand the comprehensive roles of TER94 during development.

TER94 is a highly pleiotropic AAA-ATPase associated with many essential cellular functions (Schuberth and Buchberger, 2008). Specifications of the diverse activities of TER94 and the fate of its substrates are mainly exerted by the regulatory co-factors (van den Boom and Meyer, 2018). As shown before, distinct co-factors are involved in regulating the Hh and Wg signaling pathway (Zhang et al., 2013; Reim et al., 2014). We show that one of the TER94 co-factors, p47 is required for Notch signaling activity during fly wing margin formation. The p47 co-factor was first identified as an essential component for the TER94-mediated membrane fusion pathway in yeast (Kondo et al., 1997). Subsequent studies revealed that the TER94-p47 complex plays crucial roles in cellular membrane related events, including nuclear envelop formation, ER biogenesis and Golgi assembly (Uchiyama and Kondo, 2005; Meyer et al., 2012). The molecular function of p47 is conserved from yeast to mammalian cells, but its role in animal development is not fully understood. It has been reported that the p47 homolog is essential for photoreceptor morphogenesis in *Drosophila* by affecting nuclear envelope assembly (Sang and Ready, 2002). Our finding that the TER94-p47 complex positively regulates Notch signaling in the fly wing thus reveals a novel role of TER94-p47 in development. Very recently, the fly TER94-p47 complex was shown to directly bind to and transfer poly-Ubiquitin chains for further processing (Blount et al., 2018). Indeed we observed defects in ubiquitin status in TER94 loss-of-function cells. It is reasonable to assume that the ubiquitin system is involved in TER94-p47 mediated regulation of Notch signaling.

We believe that TER94 regulates Notch protein in the signal receiving cells, but the underlying molecular mechanism is still incomplete. It has been demonstrated that Notch protein is subject to ubiquitin modification in both fly and vertebrates (Lai, 2002; Le Bras et al., 2011). The HECT-type E3 ubiquitin ligase Suppressor of deltex [Su(dx)] interacts and ubiquitinates Notch protein in *Drosophila* (Cornell et al., 1999; Yao et al., 2018). The mouse homolog of Su(dx), named Itch, adds ubiquitin chains on Notch protein in the same fashion (Qiu et al., 2000). However, the mechanism of regulation of Notch by Su(dx)/Itch remains unclear, they may function on internalization of membrane bound Notch, generation of NICD, and/or free cytoplasmic NICD (Qiu et al., 2000; McGill and McGlade, 2003). First identified in *Caenorhabditis elegans*, the *Sel-10* gene encodes an F-box containing E3 ligase that targets NICD for ubiquitination and proteasomal degradation (Hubbard et al., 1997; Deng and Greenwald, 2016). The mammalian homolog of Sel-10, FBXW7, binds to NICD to promote its ubiquitination and subsequent rapid degradation (Gupta-Rossi et al., 2001; Öberg et al., 2001; Wu et al., 2001). Despite the apparent importance of Sel-10/FBXW7 in regulation of NICD protein stability, a conclusive link between fly Sel-10/FBXW7 and Notch signaling is still missing. Two E3 ubiquitin ligases, Neuralized (Neur) and Mind bomb (Mib), have been shown to regulate a distinct subset of Notch signaling events in the signal-sending cells (Pavlopoulos et al., 2001; Lai et al., 2001; Yeh et al., 2001;Borgne et al., 2005; Lai et al., 2005). Whether the signal-receiving cells also utilize different E3 ligases to regulate distinct developmental processes is still an open question. It would be necessary to examine whether and how TER94 regulates the ubiquitination states of Notch and the relationship between TER94 and Notch E3 ligases to deepen our understanding of the regulatory roles of TER94 in Notch signaling.

## MATERIALS AND METHODS

### Fly genetics

Flies were maintained in standard medium and stocks were kept at room temperature (21–23°C). Fly crosses as shown in Figs 1, 3, 4, 5, 6C–E and 7, Figs S1, S2A–B and S3 were maintained at 25°C, while crosses related to Figs 2 and 6A–B, Fig. S2C–F and Table S1 were performed at 29°C to induce maximal Gal4 expression in the developing wing disc. Loss-of-function somatic clones were induced in the wing discs by Flp/FRT-mediated homologous recombination using the *Ubx-FLP*; *ubi-GFP*, *FRT42D*/*Cyo* stock.

The FRT42D, *TER94^k15502^*/Cyo stock (#111109) was obtained from the Kyoto Fly Stock Center. The strains of *Ter94* RNAi (#35608), *Ser8* RNAi (#41640), *Plap* RNAi (#67871), *Npl4* RNAi (#53004) and *Faf2* RNAi (#43224) were obtained from the Bloomington Stock Center. The *P47* RNAi (THO2428.N), *Casp* RNAi (THU5545), *Pngl* RNAi (THU4343) and *Ter94* RNAi (THU1058) stocks were obtained from Tsinghua Fly Center. The UAS-TER94^WT^ and UAS-TER94^AA^ transgenic flies were kindly provided by Dr. Yun Zhao (Zhang et al., 2013).

### Immunostaining and microscopy

Drosophila wing imaginal discs from third instar larvae were dissected in ice-cold 1× PBS and fixed for 15 min in 4% paraformaldehyde. Fixed tissues were washed three times with 0.1% Triton X-100 in PBS (PBST) and blocked in PBST containing 2% BSA for 1 h at room temperature. Wing discs were then incubated with the primary antibodies overnight at 4°C, followed by three washes with PBST before incubating with Alexa Fluor-conjugated secondary antibodies (1:400; Invitrogen) for 1 h at room temperature. Wing discs were dissected and mounted after washing three times with PBST. The following primary antibodies were used: mouse anti-Wingless (1:200; 4D4; DSHB), mouse anti-Notch intracellular domain NICD (1:200; C17.9C6; DSHB), mouse anti-Delta (1:200; C594.9B; DSHB), mouse anti-Cut (1:100; 2B10; DSHB), mouse anti-Ub (1:1000; FK2; Millipore), rabbit anti-K48-linkage Ub (1:100; Apu2; Millipore) and rabbit anti-K63-linkage Ub (1:100; Apu3; Millipore). Adult wings were dissected and mounted as described previously (Ren et al., 2018).

The images of adult wings were acquired with a Leica DMIL inverted microscope equipped with a QImaging QICAM Fast 1394 digital camera. The fluorescent images were acquired with a Leica SP8 confocal microscope. The figures were assembled in Adobe Photoshop CC with minor image adjustments (brightness and/or contrast).

## Supplementary Material

Supplementary information
