# Supplementary material for: The ATPase TER94 regulates Notch signaling during Drosophila wing development
Source: Biol Open. 2018 Dec 10;8(1):bio038984. doi: 10.1242/bio.038984 (PMC6361195; doi:10.1242/bio.038984)
Supplement: Supplementary information [file biolopen-8-038984-s1.pdf]

Supplemental Material

Table S1 Summary of TER94 co-factors RNAi screen

Gene Symbol	RNAi Stock	Adult phenotype	Wg/Cut expression
p47	THO2428.N	Pupae lethal	Reduced
casp	THU5545	No phenotype	No change
CG33722	THO2558.N	No phenotype	No change
CG4603	THO4863.N	No phenotype	No change
Pngl	THU4343	No phenotype	No change
CG12795	THU1504	No phenotype	No change
CG8209	BL41821	No phenotype	No change
CG8892	BL56862	No phenotype	No change
Ser8	BL41640	No phenotype	No change
Plap	BL67871	No phenotype	No change
Npl4	BL53004	No phenotype	No change
Faf2	BL43224	No phenotype	No change

All RNAi stocks were crossed with *hh-Gal4* at 29°C.

Biology Open • Supplementary information

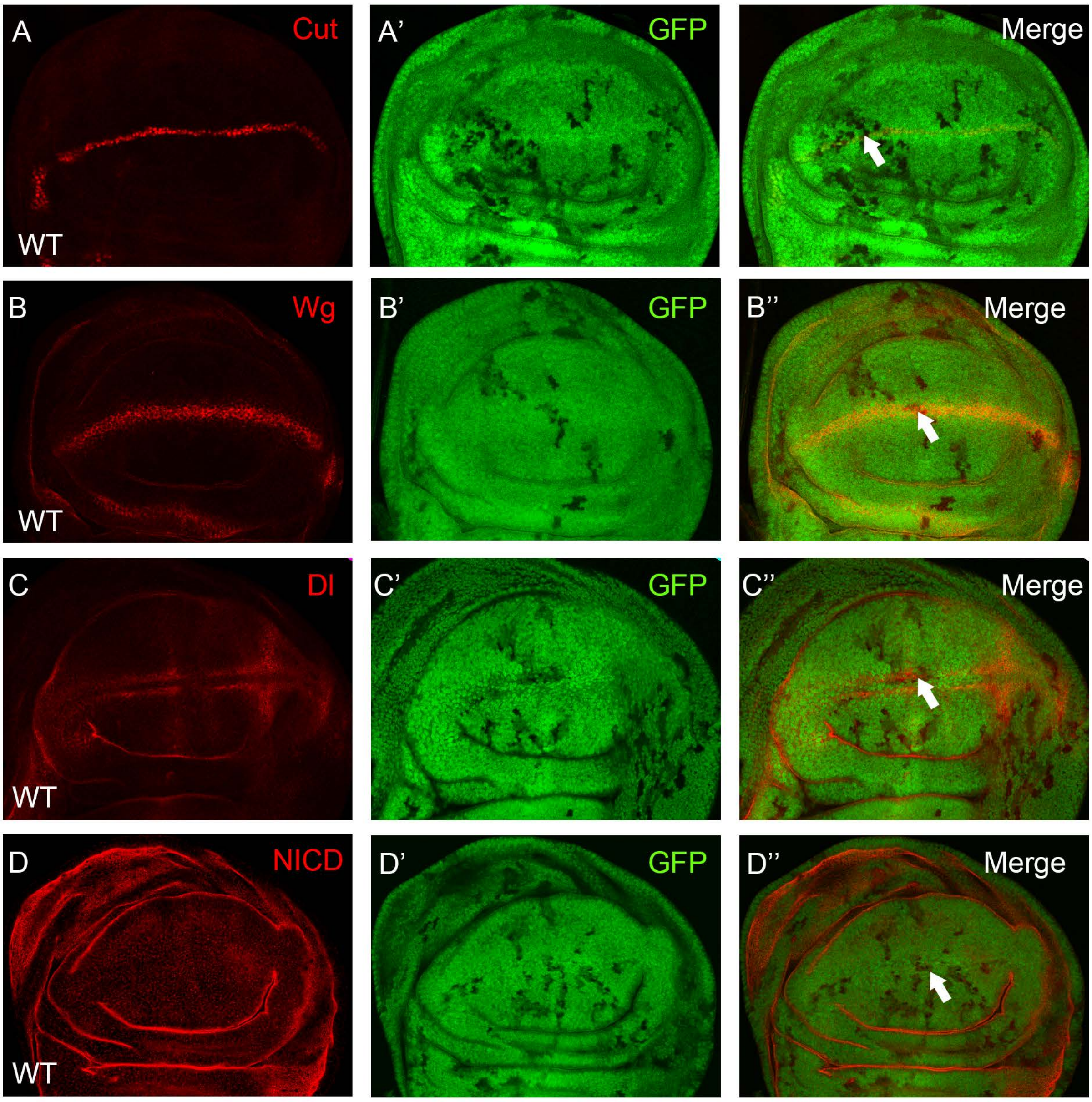

Figure S1. Expression patterns of Notch signaling components are stereotypical.

Immunostaining shows that Cut (A) and Wg (B) are expressed by cells at the D/V boundary in the pouch of wing discs. In addition, Wg are also expressed by cells at the outer ring of wing disc pouch area (B). Dl (C) and Notch (D) are expressed by all cells in the pouch with complementary patterns. Wild-type clones are induced and marked by absence of GFP. Representative clones are indicated by arrows.

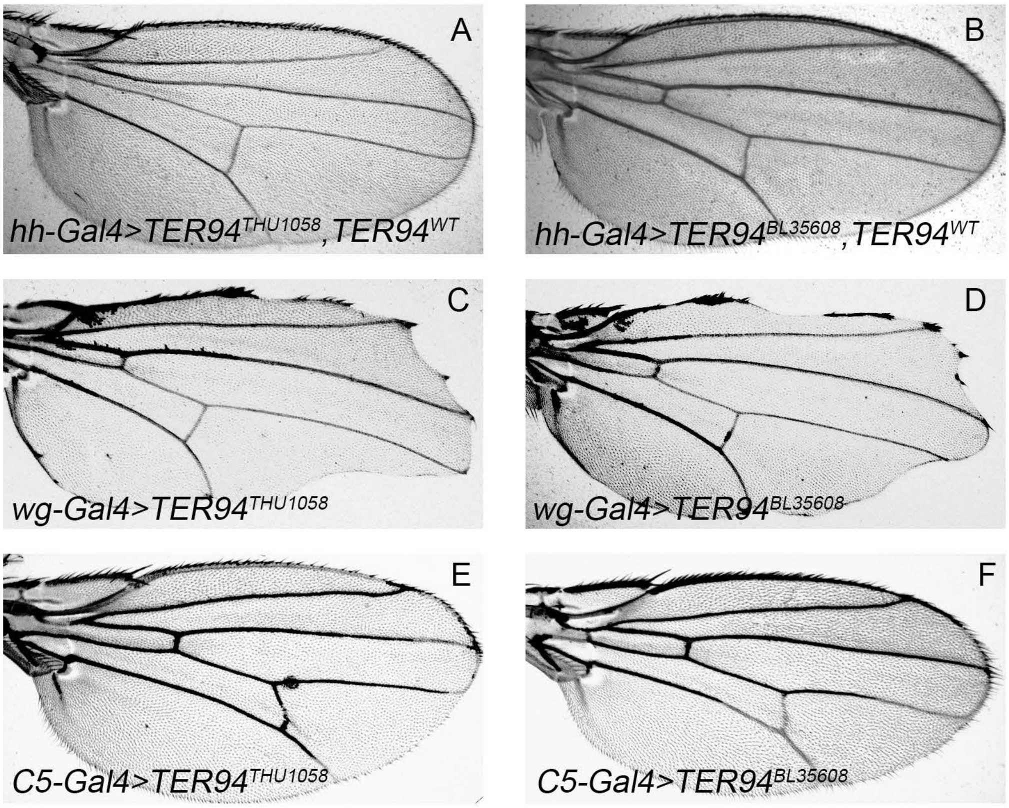

Figure S2. Knock-down of TER94 by RNAi leads to wing margin defects.

(A-B) The wing margin nicking phenotype caused by TER94 RNAi can be rescued by reintroduction of wild type TER94.

(C-D) Knock-down of TER94 in Notch signal receiving cells by *wg-Gal4* driven RNAi causes loss of wing margin tissue.

(E-F) Knock-down of TER94 in Notch signal sending cells by *C5-Gal4* driven RNAi shows no defect on wing margin formation.

Biology Open • Supplementary information

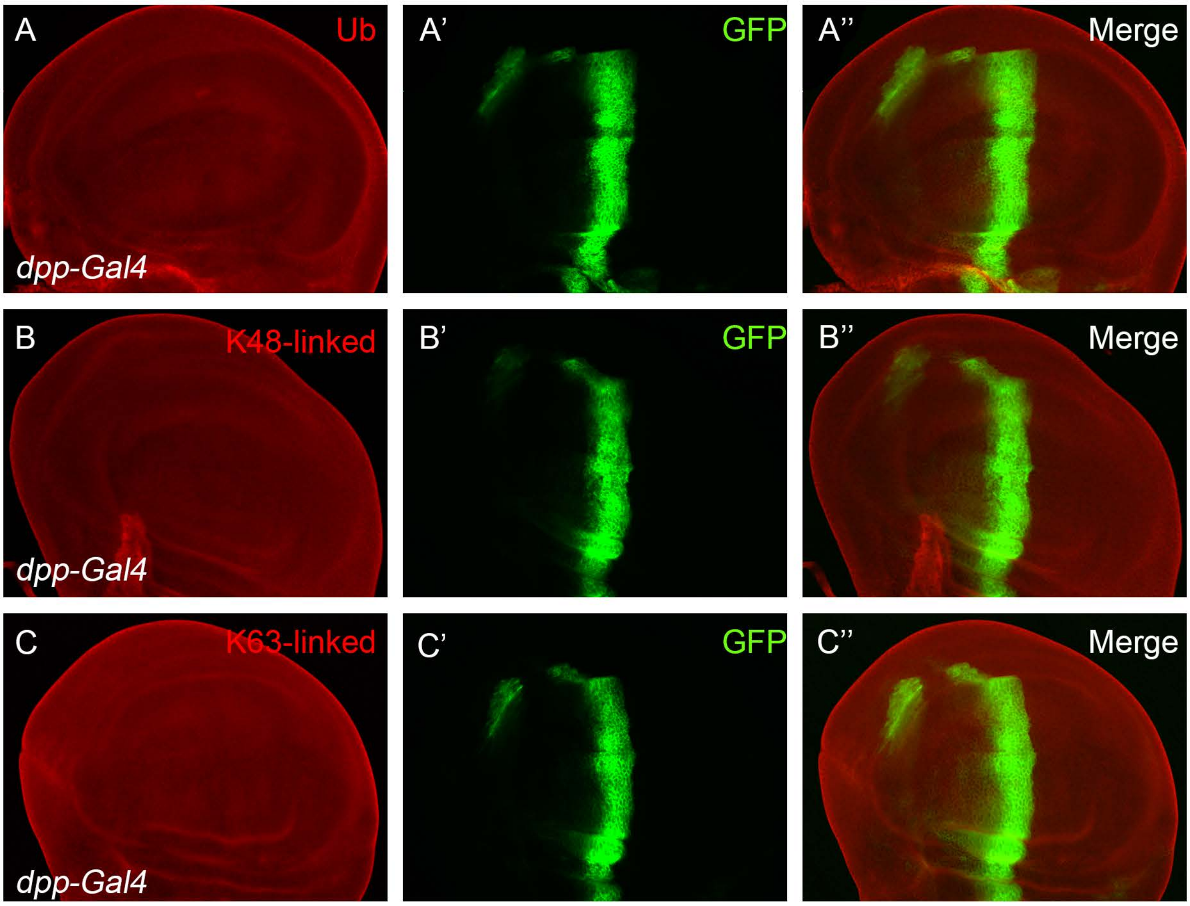

Figure S3. Ubiqutin are uniformly expressed by wing disc cells.

In the wing imaginal disc, Ub (A), K48-linked Ub (B) and K63-linked Ub (C) are uniformly distributed in all cells. GFP marks the expression domain of *dpp-Gal4*.

Biology Open • Supplementary information
